# Mapping the anterolateral ligament of the knee: a bibliometric analysis

**DOI:** 10.1186/s43019-025-00274-5

**Published:** 2025-05-09

**Authors:** Hale Öktem, Yusuf Jamil, Sinem Nur Sever

**Affiliations:** 1 Department of Anatomy, Faculty of Medicine, Atilim University, Ankara, Turkey; 2 Faculty of Medicine, Atilim University, Ankara, Turkey

**Keywords:** Anterolateral ligament, Knee stability, ACL reconstruction, Rotatory instability, Biomechanics

## Abstract

**Background:**

This study aims to evaluate research trends, key contributors, and thematic focuses in research of the anterolateral ligament (ALL) of the knee. It seeks to identify future direction for studies related to long-term clinical outcomes regarding ALL’s role in rotational stability, especially in the context of anterior cruciate ligament (ACL) injuries.

**Methods:**

A bibliometric analysis was conducted using the Web of Science (WoS) database, covering publications from 2012 to 2024 with the search term “anterolateral ligament”. A total of 942 studies were identified. Descriptive statistics summarized publication trends, authorship, institutional contributions, and citation metrics. VOSviewer software was used to analyze co-authorship network analysis, keyword co-occurrence mapping, and total citation analysis. Yearly publication and citation trends were analyzed using WoS data. Studies addressing the ALL in other body regions were excluded. Additionally, only authors with at least one publication and one citation were considered, and documents with more than 25 authors were excluded. A total citation analysis was conducted, and 24 relevant keywords with more than 5 occurrences were identified using VOSviewer.

**Results:**

Among 942 publications, 707 were original articles. Research output peaked in 2017 (125 articles). Sonnery-Cottet was the leading author (75 publications), while Universidade De São-Paulo emerged as the top institution (57 publications). Key journals included *Arthroscopy: Journal of Arthroscopic and Related Surgery* (143 articles) and *The American Journal of Sports Medicine* (131 articles). Keywords such as “anterior cruciate ligament”, “reconstruction”, and “rotational stability” dominated, reflecting a focus on ACL injury management. The top ten cited studies accrued 3,86 citations, with Claes et al.’s anatomical study leading (621 citations). Of the 942 ALL-related articles in WoS, 381 focused on anatomy (11,278 citations) while 814 addressed reconstruction (17,048 citations). Keyword trends shifted from anatomical to clinical terms, with anatomy declining and stability, injury, and outcomes gaining prominence from 2021 to 2024.

**Conclusions:**

This bibliometric analysis underscores the growing interest in ALL research, peaking between 2016 and 2017. While foundational studies on ALL anatomy and biomechanics appear saturated, future research should prioritize clinical outcomes in terms of failure rate, reoperation, the long-term efficacy of ACL–ALL reconstruction, and advancements in imaging techniques.

## Introduction

The anterolateral ligament (ALL) of the knee, a crucial structure for maintaining rotational stability, has recently reemerged as a significant focus in orthopedic and sports medicine [1]. Together with the anterior cruciate ligament (ACL), it is responsible for controlling internal tibial rotation and resisting pivot-shift forces, which are critical in preventing rotatory knee instability [2, 3]. Biomechanical studies have demonstrated that ACL injuries often result in concomitant damage to the ALL, particularly in cases of high-grade rotatory instability [4]. This has raised questions about the sufficiency of isolated ACL reconstruction in restoring full knee stability, leading to increased interest in combined ACL and ALL reconstruction techniques aimed at reducing graft failure and improving clinical outcomes, especially in athletes and individuals with high physical demands [5]. Despite its importance, research on the ALL has lagged behind that of other knee structures, resulting in variability in reported anatomy, biomechanical function, and treatment approaches [6]. As research shifts toward this ligament, bibliometric analysis becomes essential to comprehensively assess the research landscape surrounding the ALL.

A bibliometric analysis is a quantitative method used to evaluate scientific literature [7]. In the context of ALL, this analysis is designed to map research trends by analyzing publication growth over time. It identifies prolific authors and leading institutions contributing to ALL research and analyzes keywords to highlight recurring themes. These themes include the ALL’s relationship with ACL injuries, rotatory instability, biomechanics, and reconstruction techniques [8]. By evaluating scientific literature quantitatively, a bibliometric analysis not only charts the course of past research but also pinpoints knowledge gaps and guides future research directions. This comprehensive approach significantly enhances the understanding and management of knee instability and ligamentous injuries among clinicians, underscoring the critical contribution of bibliometric analysis to the evolution of orthopedic research.

Such analysis is essential to map research trends, identify prolific authors and leading institutions, and highlight recurring themes that include the ALL’s relationship with ACL injuries, rotatory instability, biomechanics, and reconstruction techniques [7, 8]. By evaluating scientific literature quantitatively, a bibliometric analysis not only charts the course of past research but also identifies gaps and guides future research directions, thereby enhancing clinicians’ understanding and management of knee instability and ligamentous injuries.

Given the existing gaps in clinical outcomes related to ALL research, particularly regarding failure rates, reoperation, long-term efficacy of ACL–ALL reconstruction, and advancements in imaging techniques, this study is poised to address these critical issues. This bibliometric analysis aims to answer how understanding of the anterolateral ligament has evolved over time and to identify the primary clinical outcomes and gaps that future research must address to enhance the management of knee stability.

## Materials and methods

This bibliometric analysis conducted in October 2024 focused on publications related to the anterolateral ligament (ALL) of the knee. Data were collected from the Web of Science (WoS) database, which is known for indexing high-impact journals. The search was conducted using the keyword “anterolateral ligament”. The period included publications from 2012 to 2024. All relevant document types, including original articles, review articles, meeting abstracts, and editorial materials, were initially considered. Studies specifically addressing the anterolateral ligament, or its anatomical, clinical, and biomechanical aspects, in the knee were included. Articles focusing on other anterolateral ligaments of the body were all excluded. Articles in all languages were included in the study to allow for enhanced research all over the world.

The search yielded a total of 942 publications, among which articles were filtered on the basis of the inclusion criteria. Information regarding publication years, authors, journals, countries, institutions, and keyword frequencies were extracted using VOSviewer (version 1.6.20, Leiden University) for bibliometric analysis. Articles were categorized into relevant WoS categories to visualize the scope of research fields and trends over time. To ensure a reliable bibliometric analysis regarding the ALL, authors with a minimum of one document and one citation were included, while the rest were excluded. Of the 278 authors, 250 met this threshold.

### Statistical analysis

Data extracted from the Web of Science database were subsequently imported into VOSviewer for detailed visualization and mapping. Within VOSviewer, maps for each “type of analysis” were generated from a bibliometric database file, employing a “full counting method” to ensure comprehensive data inclusion. Each analysis type was aligned with its corresponding “unit of analysis” to reflect the research landscape. Documents featuring more than 25 authors were excluded from the analysis, ensuring focus on the most collaborative and impactful research.

Descriptive statistics, including frequency distributions and percentages, were used to summarize the data. Relationships between authors, journals, and institutions were visualized through co-authorship network analysis and keyword co-occurrence maps generated by VOSviewer. A citation report file was exported from WoS, which was then used to generate charts and analyze trends over a yearly period. These data facilitated a comparative analysis of publications and citations per year. Inclusion criteria comprised a total citation analysis with self-citations included.

The minimum keyword occurrence threshold was set to five, and a keyword map was generated using VOSviewer. The analysis identified 24 distinct, relevant keywords across the selected articles.

## Results

A total of 942 publications were obtained through literature review. The most popular document types were as follows: articles (707), review articles (106), editorial material (77), letters (29), meeting abstracts (15), and corrections (7). Most of them were original articles (75.05%). Of these articles, 98.2% (925) were written in English. Other articles were written in German (11), Russian (3), Czech (1), Spanish (1), and Turkish (1). The first study on the anterolateral ligament of the knee was in 2012. This publication was written by Vincent and was published in the journal *Knee Surgery, Sports Traumatology, Arthroscopy*.

The highest number of publications on the anterolateral ligament was in 2017 (125 studies), and the lowest number in 2012 (1 study).

**Fig. 1:**
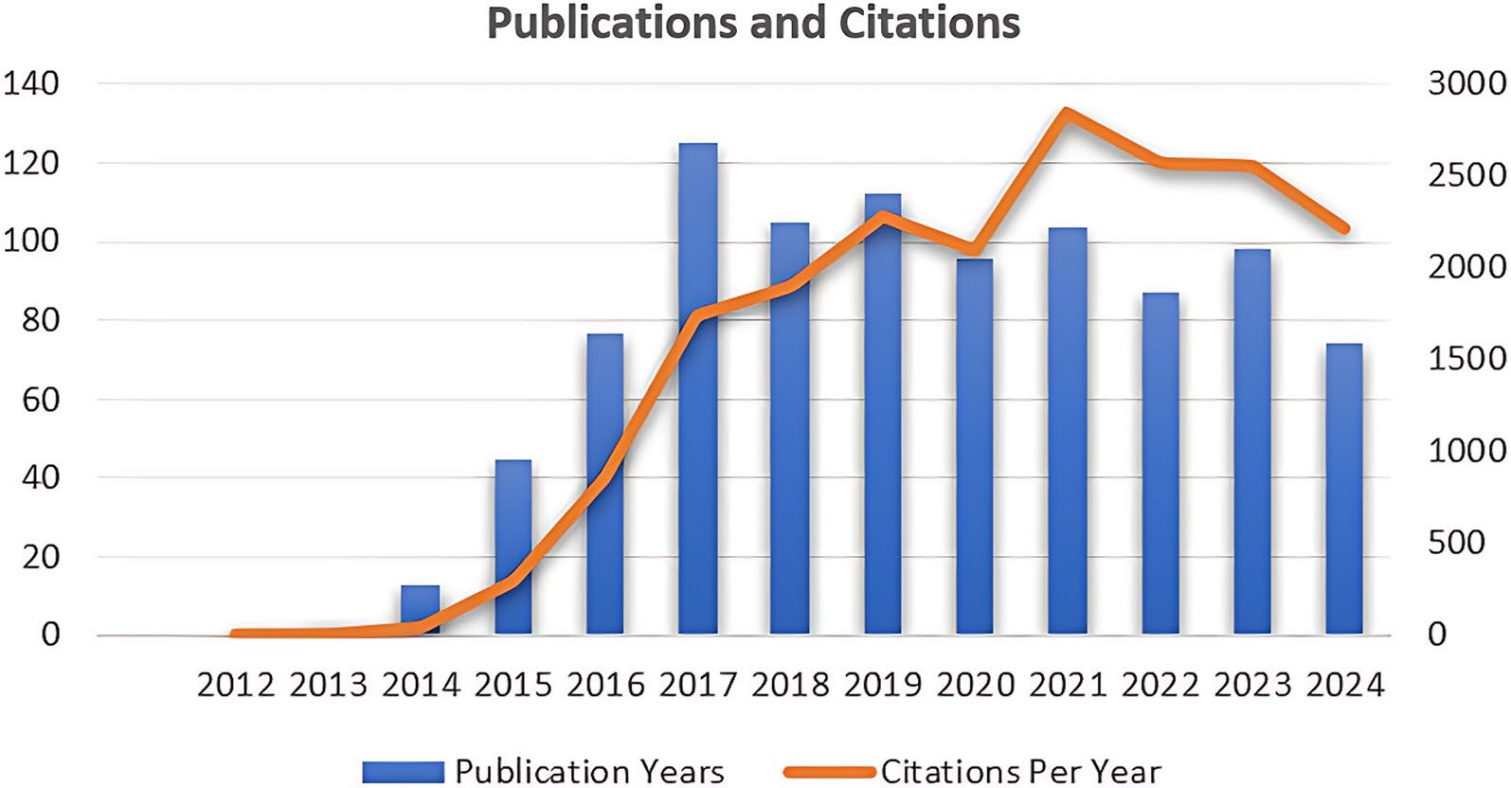
Trends in publications and citations (2012–2024)

With an average of 72.23 articles per year (mean ± standard deviation of 42.99), the number of publications from 2016 to 2024 exceeded this average. Additionally, following the first article on the anterolateral ligament of the knee, published in 2012, there was an exponential increase in original article publications until 2017. The trends also indicate a slight decline in the rate of publications on the ALL since 2021. Figure 1 shows the distribution of articles on the anterolateral ligament by year, as well as the average citations per year.

### Active authors and institutions

**Fig. 2:**
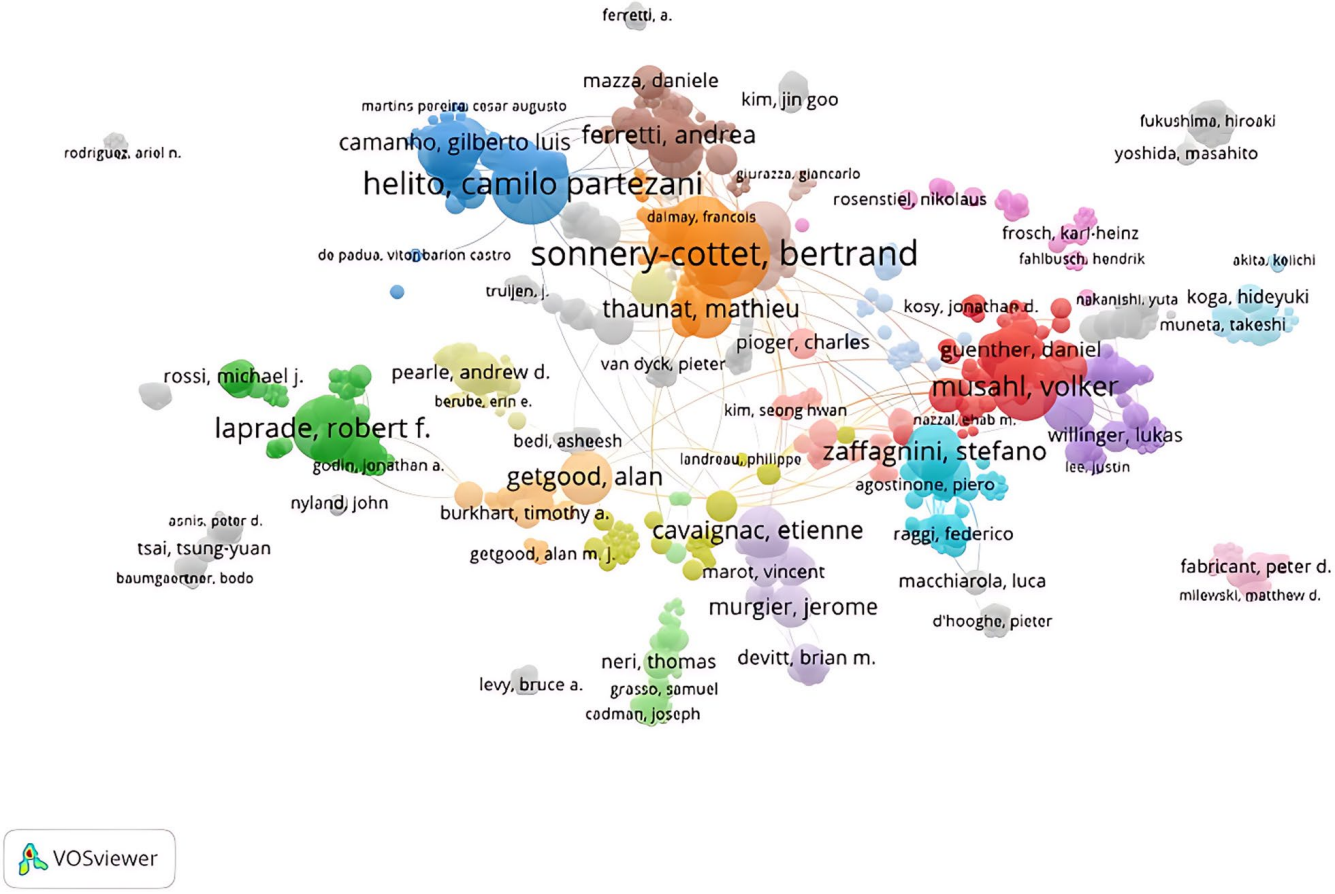
Network visualization map for cluster analysis of active authors researching ALL

The top ten authors who produced the highest number of articles related to the anterolateral ligament of the knee were as follows: Sonnery-Cottet (75), Helito (62), Saithna (44), Musahl (39), Laprade (35), Zaffagnini (34), Monaco (29), Ferretti (28), Thaunat (28), and Vieira (28) (Fig. 2).

The top ten institutions that produced the highest number of articles concerning the anterolateral ligament were Universidade de São Paulo (57), Pennsylvania Commonwealth System of Higher Education PASSHE (50), University of Pittsburgh (49), Hôpital Privé Jean Mermoz (48), Centre Orthopédique Santy (35), Western University of Western Ontario (35), Sapienza University Rome (32), Hospital Sirio Lebanese (28), Azienda Ospedaliera Sant’Andrea (27), and Imperial College London (24).

### Popular WoS categories and active research areas

The anterolateral ligament is covered by 37 WoS categories. The number of articles was refined according to the most common and relevant categories to the anterolateral ligament of the knee, with the top ten as follows: orthopedics (749), sport sciences (505), surgery (365), radiology nuclear medicine medical imaging (52), medicine general internal (40), anatomy morphology (22), engineering biomedical (15), rheumatology (10), medicine research experimental (8), and multidisciplinary sciences (8).

Furthermore, the most popular 10 active research areas, out of a total of 34 fields, were as follows: orthopedics (749), sport sciences (505), surgery (365), radiology nuclear medicine medical imaging (52), general internal medicine (45), anatomy morphology (22), engineering (19), rheumatology (10), research experimental medicine (8), and science technology other topics (8).

### Productive countries

**Fig. 3A:**
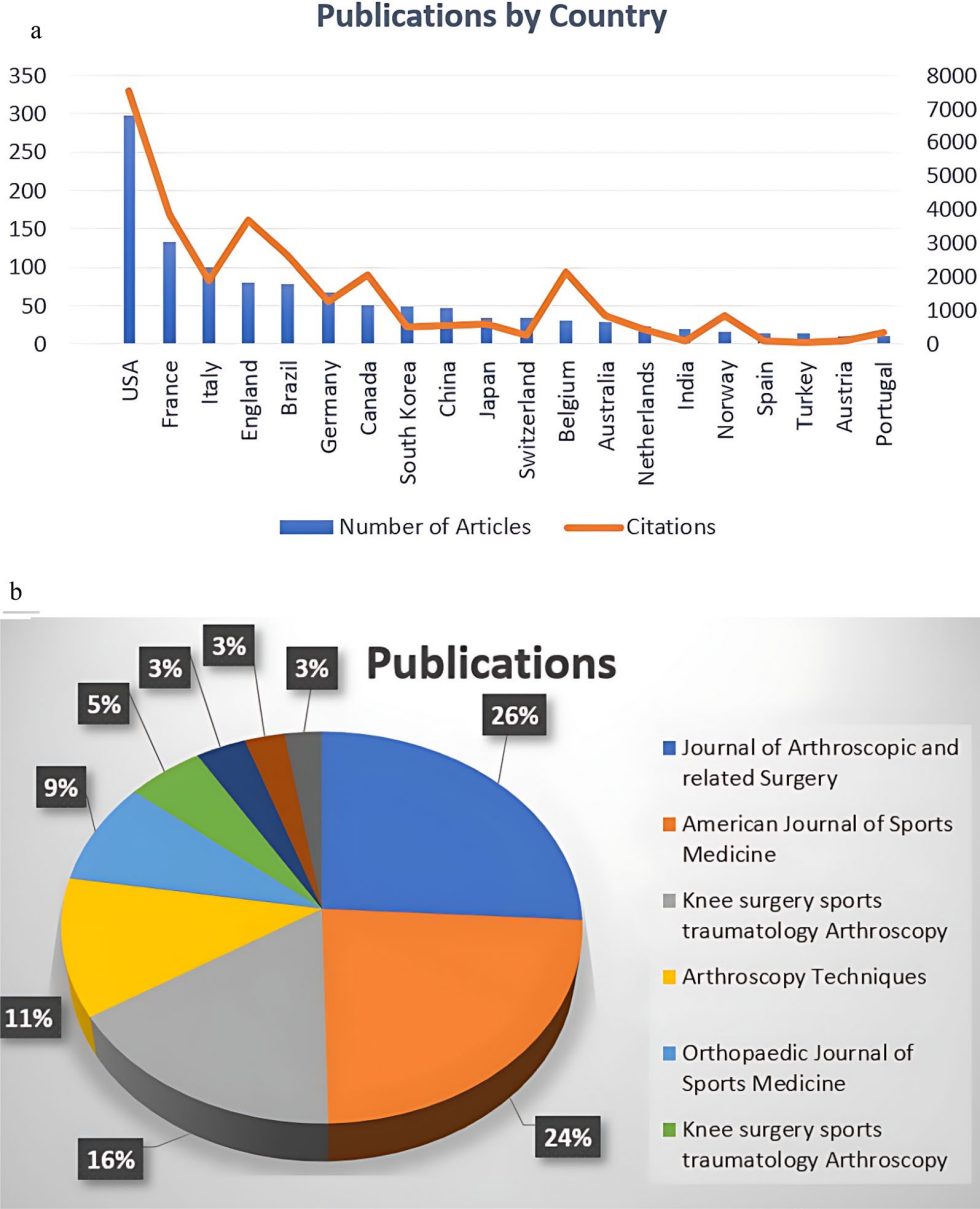
Distribution of the most productive countries. **B** Distribution of publications on ALL research by journal

A total of 60 countries contributed to the publications. The ten most active countries were the USA (298), France (134), Italy (101), England (81), Brazil (78), Germany (68), Canada (51), South Korea (49), China (48), and Japan and Switzerland, each with 34 publications (Fig. 3A).

### Active journals

Articles were published in a total of 153 journals, 16 of which published more than ten articles (Fig. 3B). Most articles were published in *Arthroscopy: The Journal of Arthroscopic and Related Surgery* (143) (ISSN 0749–8063), which provides a platform for high-quality research, reviews, and innovations focused on minimally invasive surgical techniques and advancements in arthroscopy, orthopedic surgery, and related procedures. This journal published the largest number of original papers, followed by the *American Journal of Sports Medicine* (131) and *Knee Surgery, Sports Traumatology, Arthroscopy* (91).

## Citation analysis

A total of 19,408 citations were recorded in the WoS database for studies on the anterolateral ligament between 2012 and 2024. The ten most cited studies, along with the number of citations and the average citation count per year, are given in Table 1.

**Table 1:** The top ten most cited studies and their number of citations

Article	Number of citations	Average citations per year
Claes et al.	621	51.75
Sonney-cottet et al.	332	33.2
Getgood et al.	330	66
Sonnery-Cottet et al.	315	39.38
Dodds et al.	308	28
Vincent et al.	299	23
Kennedy et al.	274	27.4
Parsons et al.	248	24.8
Kittl et al.	232	25.78
Getgood et al.	225	37.5

### Citation analysis according to themes

Out of the 942 articles on ALL indexed in WoS, 381 included anatomy as a key topic, accounting for 11,278 citations. In contrast, 814 studies focused on reconstruction, whether related to the ALL, ACL, or ALL–ALL combined, contributing a total of 17,048 citations, exceeding those related to anatomy.

### Keyword analysis

**Fig. 4:**
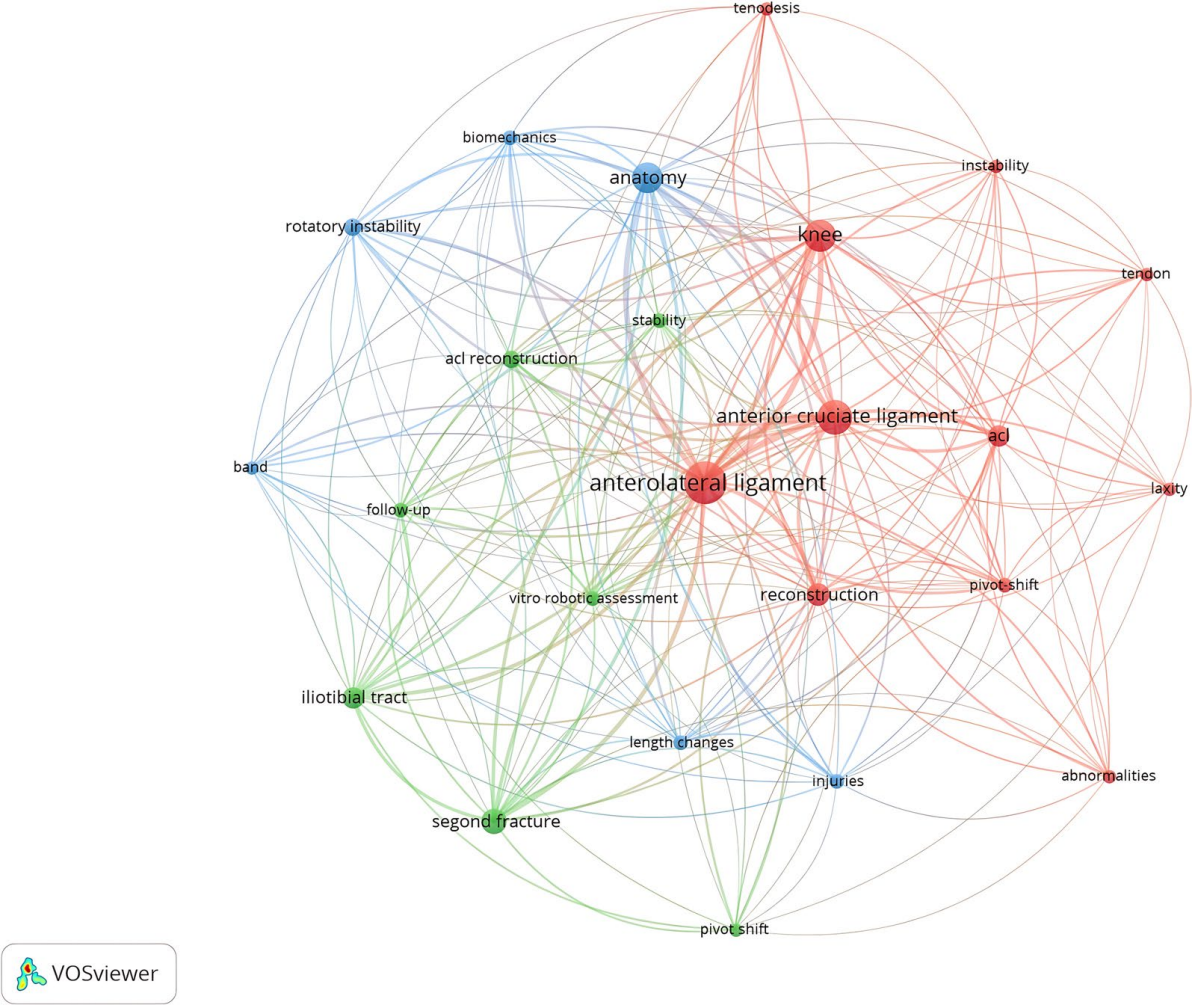
Keyword analysis of ALL

Keywords are crucial in research articles as they enhance discoverability, streamline searches, and ensure that work reaches the appropriate audience. Repetition of words across multiple articles suggests their significance in the field of research [9]. To accurately reflect the study’s core themes and focus areas, we implemented a threshold of a minimum of five occurrences for a keyword. Of the 184 keywords, 24 met this threshold, with the most common keywords being “anterior cruciate ligament”, “knee”, “anatomy”, “Segond fracture”, “reconstruction”, “iliotibial tract”, “ACL”, “ACL reconstruction”, and “rotatory instability” (Fig. 4). The most popular keyword used across all research was “anterior cruciate ligament”. Since the two ligaments are closely related in knee stability, studies on the ALL often overlap with ACL research, as both structures are frequently discussed together in the context of knee injuries, reconstruction techniques, and joint biomechanics. The ACL’s prominence in orthopedic research makes it a natural reference point in studies on the ALL [10]. It is important to note that the least common keywords were “laxity”, “tendon”, “tenodesis”, and “abnormalities”. Although the term “laxity” was the rarest, injury to the ALL may increase the pathological laxity associated with an ACL injury [11].

### Evolution of common keywords over time

An analysis of keyword trends revealed a shift in focus from anatomical to clinical terms over time. The most common keywords were categorized into three distinct time periods.

Between 2012 and 2016, a total of 381 keywords were identified, with only 41 words occurring a minimum of 5 times. The top 10 keywords during this period were “ALL” (79), “anatomy” (78), “ACL” (60), “knee” (41), “iliotibial tract” (38), “Segond fracture” (36), “ACL reconstruction” (36), “pivot shift” (27), and “follow-up” (23). Notably, anatomy was among the most dominant keywords.

In the following period, 2017–2020, the number of unique keywords increased significantly to 982, with 156 meeting the criteria. The top keywords in this timeframe were “ALL” (281), “ACL” (229), “knee” (178), “anatomy” (142), “reconstruction” (119), “ACL reconstruction” (104), “ACL” (89), “Segond fracture” (75), “pivot shift” (58), and “follow-up” (58). While anatomy remained a key term, there was a notable increase in the use of clinical terms such as “reconstruction” and “follow-up”.

From 2021 to 2024, 1,044 unique keywords were identified with 134 words meeting the threshold. The top keywords during this period were “ALL” (242), “ACL” (185), “knee” (131), “ACL reconstruction” (85), “reconstruction” (81), “ACL” (75), “anatomy” (70), “stability” (59), “injury” (62), and “outcomes” (55). The prominence of anatomy declined further, while clinical terms such as stability, injury, and outcomes gained more relevance.

## Discussion

Bibliometric studies conducted within the scientific field help guide future studies by analyzing the numerical data of scientific publications [12]. This study represents the first comprehensive bibliometric analysis of the ALL and offers detailed insights into the research trends, authorship, and institutional contributions related to this ligament.

Original studies in the field introduce new perspectives, employ diverse methodologies for in-depth analysis, and contribute to the advancement of existing literature [13]. A review of ALL studies indicates that 75.05% are original articles, reflecting active knowledge production and continuous scientific engagement in the field.

Considering that English is the primary language of scientific and academic writing internationally, 98.2% of studies in the field of ALL have been published in English [14]. This widespread use of English facilitates the exchange of knowledge, ensuring that research findings are more effectively accessed, cited, and integrated into the global scientific field.

The main findings of this bibliometric analysis highlight a notable growth in publications focused on the ALL of the knee, particularly from 2016 onward, with a peak observed in 2017. This reflects the renewed interest in the ALL after its clinical and biomechanical significance was better understood, especially in relation to ACL injuries and knee stability [15, 16]. The steady increase in research activity from 2012 to 2017 can be attributed to a growing understanding of the ALL’s importance in knee stability and its impact on ACL reconstruction outcomes. This trend aligns with the broader interest in improving surgical methods to address knee rotational instability, a common complication following isolated ACL reconstruction [17].

An analysis of studies over the years reveals a decline in the number of publications on ALL since 2021. This decrease may be attributed to a shift in research focus on more prevalent conditions of the knee, a reduction in ligament-related injuries, or the lack of new surgical techniques or clinical anatomical advancements in this area. Additionally, the standardization of ALL treatment approaches and surgical techniques may have led to a more established clinical literature, prompting researchers to explore new innovative fields.

Multidisciplinary studies involve integrating knowledge from different fields to address a problem or research question. This approach enables a more comprehensive analysis by considering multiple perspectives. Each discipline evaluates the same topic from its own viewpoint, offering diverse solutions that enhance problem-solving efficiency and effectiveness [18]. An examination of popular WOS categories reveals that research on ALL spans various fields, including orthopedics, sports sciences, surgery, radiology, nuclear medicine, medical imaging, general internal medicine, anatomy and morphology, biomedical engineering, rheumatology, experimental medicine, and multidisciplinary sciences. This indicates that work on the ALL is not only limited to orthopedics but is relevant to numerous scientific disciplines, highlighting the potential for multidisciplinary research in this area. 

Global interest in a research field reflects its scientific significance and the widespread attention it receives from researchers worldwide. This suggests that the findings and advancements in the field are not limited to a specific region or country but hold relevance for the international scientific community, thereby contributing to a broader and more impactful body of knowledge [19]. An analysis of the most productive countries in ALL research reveals active contributions from nations such as the USA, France, Italy, England, Brazil, Germany, Canada, South Korea, China, Japan, and Switzerland. This widespread engagement underscores ALL as a globally relevant research topic, attracting interest from diverse scientific communities around the world. At the institutional level, Universidade de São Paulo leads in ALL research, with 57 publications, reflecting Brazil’s strong contribution to the development of ALL surgical techniques [19]. Other prominent institutions include the University of Pittsburgh and Santy Orthopedic Centre, both known for their clinical and experimental work on knee ligament injuries. This international collaboration underscores the global relevance of ALL research, with the USA and France emerging as leaders in this domain [5].

Identifying active journals in ALL research offers significant advantages for both researchers and academic professionals. These journals serve as key sources for the latest studies, providing access to the most recent findings and developments within the scientific community [20]. This study also presents the active journals in the field of ALL. The results indicate that *Arthroscopy: The Journal of Arthroscopic and Related Surgery* has emerged as the most influential journal in the field, contributing 15% of the total publications. This journal’s high impact factor, coupled with its focus on sports-related injuries and arthroscopic techniques, explains its central role in disseminating ALL research [6, 21]. Other prominent journals include *The American Journal of Sports Medicine* and *Knee Surgery, Sports Traumatology, Arthroscopy*, which together published nearly 24% of the total papers. Among the most cited studies, Claes et al.’s study on the “Anatomy of the anterolateral ligament” of the knee has received 622 citations, marking it as foundational in this research area [15]. This may be a result of most articles conducting research based on an anatomical foundation.

The number of citations is a key metric that indicates how frequently a scientific paper is referenced. It provides insight into a research study’s or author’s academic impact, influence, and visibility [22]. Highly cited papers typically represent foundational and most influential studies in a given field [23]. This study highlights the most cited papers on ALL. Focusing on these publications allows researchers in the field to establish a strong foundation on the topic. Additionally, analyzing the key themes and findings within these studies can help identify existing gaps and areas that require further research. The top ten most cited articles collectively garnered 3186 citations, emphasizing the importance of biomechanical and clinical studies in advancing understanding of the ALL. This high citation count for key papers highlights the strong relevance of the ALL in contemporary orthopedic practice.

An analysis of citation trends by theme reveals that studies on “ALL, ACL, or ALL–ALL combined” have received more citations than those focused on anatomy. Given that clinical research has a more direct impact on improving healthcare services, these studies may have attracted greater interest. The higher citation rates of clinically focused papers compared with those in basic medical sciences suggest an increasing emphasis on practical applications and clinical findings in medical research.

Prolific authors are researchers who have extensively explored key topics and research areas within their field. Their work can provide valuable insights, inspire new ideas, and introduce innovative research questions or methodological approaches [24]. Regarding individual authorship, Sonnery-Cottet emerged as the most prolific contributor to ALL research, with 71 articles. Their research primarily focuses on combined ACL and ALL reconstruction techniques, which have gained widespread attention owing to their success in improving patient outcomes. Other leading authors include Helito and Claes, who have also contributed significantly, particularly in biomechanical and anatomical studies.

Keywords help facilitate the discovery of relevant sources in the literature, making it easier to identify key studies. They also provide insight into underresearched topics and highlight areas where more studies have been conducted [25]. This enables the identification of new research opportunities and gaps in the field. Keyword co-occurrence analysis further reveals important trends in ALL research. The most frequent keywords include “anterior cruciate ligament”, “reconstruction”, “rotational stability”, and “knee”, indicating that ALL research is often discussed in the context of ACL injuries. This is unsurprising, as the ALL is thought to play a critical role in augmenting rotational stability following ACL reconstruction [4]. Other frequently occurring keywords include “biomechanics”, “surgery”, and “magnetic resonance imaging (MRI)”, highlighting the central focus on surgical techniques, anatomical studies, and diagnostic advancements in the field. An analysis of keywords over the years reveals a shift in focus from anatomy as a primary area of interest to a growing emphasis on clinical studies. This trend suggests an increase in research related to clinical trials and treatment methods. Additionally, this shift may be linked to technological advancements, such as improvements in imaging techniques and the need for patient specific approaches. As new treatment methods and clinical applications are integrated into the scientific literature, the use of clinical terminology has likely become more prevalent.

The presence of terms such as “outcomes”, “failure”, and “reoperation” in later publications suggests a shift in focus from anatomy and biomechanics toward the long-term effectiveness of ALL reconstructions. This trend reflects the growing interest in patient-centered outcomes, particularly in preventing re-injury and ensuring the longevity of surgical repairs.

The gap in long-term, high-quality clinical data is a significant area for future exploration. While short-term studies demonstrate the efficacy of combined ACL and ALL reconstruction, there remains a need for robust data on failure rates, reoperation frequencies, and long-term functional outcomes in diverse populations. The use of advanced imaging modalities such as three-dimensional (3D) MRI and the role of robotic-assisted surgery are also emerging areas of interest that could shape the next phase of ALL research. 

This bibliometric analysis is subject to several limitations. First, the data were exclusively sourced from the Web of Science database, potentially omitting relevant publications indexed in other databases such as PubMed or Scopus, which may lead to incomplete representation of the research landscape. The study also focused on quantitative metrics such as publication count and citation analysis, which, while useful, do not account for the quality or clinical relevance of individual studies. However, this study encompasses all works indexed in WOS without prioritizing impact factors, meaning that low-impact studies have not been excluded. Furthermore, there is a lack of long-term clinical outcome data, particularly in relation to the efficacy of ALL reconstructions, highlighting the need for further clinical research to address this gap.

This analysis not only maps the current research landscape surrounding the ALL but also identifies future directions. The growing recognition of the ALL’s clinical significance, especially in conjunction with ACL injuries, suggests that ongoing studies will likely focus on refining surgical techniques and reducing the risk of ACL graft failure. Furthermore, biomechanical research on the ALL’s role in rotatory knee instability continues to gain momentum, potentially setting the stage for future breakthroughs in orthopedic surgery and rehabilitation. Nevertheless, this analysis provides a robust overview of the ALL research landscape and offers valuable guidance for future investigations into this increasingly important area of knee ligament research.

## Data Availability

The datasets used and/or analyzed during the current study are available from the corresponding author on reasonable request.

## Abbreviations

ALL: Anterolateral ligament
ACL: Anterior cruciate ligament
WoS: Web of Science
MRI: Magnetic resonance imaging
3D: Three-dimensional
